# Feeling of self-worth in healthy premenopausal women—relationships with menstrual cycles and ovulation over 1-year in the prospective ovulation cohort

**DOI:** 10.1371/journal.pone.0327539

**Published:** 2025-08-12

**Authors:** Nahid Shirazian, Sonia Shirin, Dharani Kalidasan, Jerilynn C. Prior

**Affiliations:** 1 Department of Medicine, Endocrinology and Metabolism Division, University of British Columbia, Vancouver, British Columbia, Canada; University of Hawai'i at Manoa, UNITED STATES OF AMERICA

## Abstract

Self-Worth is an over-arching evaluation of a person’s sense of individual value. Self-worth, however, is an underappreciated concept. It has rarely been reported related to physiological data; we found no studies related to the menstrual cycle or ovulation. It is also unclear if Self-Worth is a stable trait or a variable state. We sought to discover if “Feeling of Self-Worth” (as recorded daily) was related to cycle phases and ovulation of spontaneous menstrual cycles (without hormonal contraception) in healthy premenopausal women over one-year in data from the Prospective Ovulation Cohort. Participating women were invited to complete the Menstrual Cycle Diary© (Diary©) daily; it describes cycle-related and other comprehensive everyday life experiences including negative moods as well as changes from each person’s usual Feelings of Self-Worth. Women recorded their Feeling of Self-Worth on a 5-level ordinal scale as a letter translated to a number originally centered on their usual feeling (U = 3) with two higher and two lower (letters) levels. The validated Quantitative Basal Temperature© (QBT©) method was used to assess ovulation and luteal lengths. Fifty-three healthy, community dwelling, normal-weight, non-smoking premenopausal women mean age 33.9 (95% CI 32.4, 35.5) years old were studied. All participants were first required to document two consecutive normal-length (21–36 days) and normally ovulatory (luteal length ≥10 days) cycles before enrolment. Each participant completed the Diary© and QBT© records daily over a mean of 13 cycles (minimum = 8). During the study, cycle lengths were mean 28.04 (95% CI 27.80, 28.28) days with 71% of all cycles being normally ovulatory, 26% having short luteal phases (SLP, LL < 10 days) and 2.6% being anovulatory. Results in all women and all cycles showed that the mean annual Feeling of Self-Worth was 3.01 (95% CI 2.94, 3.09), thus very tightly related to their usual Feeling of Self-Worth. There were only small, inconsistent differences between Feeling of Self-Worth in the follicular versus luteal phases comparing normally ovulatory versus all ovulatory cycles (including those with short luteal phases). Analysis of Self-Worth within the 46 women having both normally ovulatory and ovulatory disturbed cycles (short luteal and anovulatory) showed that it was slightly lower in these women’s normally ovulatory cycles (*P* = .03). Principal Components Analysis of all Diary© data showed that Feeling of Self-Worth was positively related to Interest in Sex and Feeling of Energy (together explaining 9% of all variance). In addition, Feeling of Self-Worth had a significant, negative loading on the Negative Mood Factor (that explained 14.2% of total variance). These data suggest that Feelings of Self-Worth in this comprehensive menstrual cycle and ovulation dataset in healthy women were not related menstrual phases and ovarian hormone levels.

## Introduction

The current investigation is part of a larger study: “Menstrual Cycle and Ovulation-Related Experiences in Healthy Menstruating Premenopausal Women, the 1-year Prospective Ovulation Cohort (POC)” [1]. Archived daily Menstrual Cycle Diary© (Diary) data collected prospectively during a primary published study [2] was used for the 1-year POC in the 53 women with at least eight cycles of data. In the following investigation, we describe Feeling of Self-Worth in this healthy, premenopausal cohort, investigate its relationship with spontaneous menstrual cycle phases and ovulation characteristics, and explore its association with other experiences as also recorded in the daily Diary [3]. Estradiol levels start low on the first days of flow and rise over 9–14 + days to 220% above that level. By contrast, progesterone levels remain low from flow until following ovulation when progesterone rises to a plateau that is 1400% above its flow-related baseline. Whether and how these hormonal changes influence women’s feelings continues to be controversial [4].

Individuals want to feel that they are worthy and valuable [5]. The American Psychological Association says, “self-worth (is) defined as an individual’s evaluation of themself as a valuable, capable human being deserving of respect and consideration. Positive feelings of self-worth tend to be associated with a high degree of self-acceptance and self-esteem” [6]. Some early clinical psychologists like Rogers in his “*Development of Conditions of Worth*” discussed self-worth as a unique psychological construct [7]. Later, Harter in his book, “*The Construction Of The Self*”, used “self-esteem” and “self-worth” interchangeably [8]. Harter believed that self-worth— was an overall evaluation of one’s worth or value as a person [8]. Rogers [7] proposed that individuals, based on their positive achievements and experiences, would develop conditions of self-worth; however if they only received positive regard, no condition of self-worth would develop. Although self-worth is an under-appreciated concept, low self-worth has a central role in people experiencing different psychiatric disorders. More specifically, feeling of “worthlessness” is one of nine criteria the DSM-5 used to diagnose Major Depressive Disorder (MDD) [9].

It is also unclear from the literature whether self-worth is a stable **trait**, or a variable **state.**

Trzesniewski KH et al. [10] investigated the level of stability of self-worth over time by performing two studies: study 1, a meta-analysis of 50 published articles; study 2 analysis of longitudinal data from 4 large studies. They concluded that self-worth seemed similar to other *stable personality traits* throughout much of the life span, only diverging in old age [10]. On the other hand, in some studies, experimental manipulations resulted in temporary changes in self-worth. For example, Markus and Kunda [11], showed temporary variability of self-worth after exposing 40 female university students to a socially challenging manipulation. The manipulation led students to find themselves to be either different from or unique related to others (decreasing self-worth) or similar to others (increasing self-worth). Individuals who were led to feel unique were disturbed by this knowledge and viewed the state of uniqueness as negative and undesirable, but the state of similarity to others as positive and desirable. Students who were made to feel similar to others and responded in exactly the opposite way. Researchers conclude the self-worth as a concept is mostly stable but can also be malleable. Although all participants of this study were women, researchers did not access anything about the menstrual cycle phase in which the study was conducted and also all were university students thus decreasing applicability.

Different scales have been developed to study the stability and variability of self-worth. The Rosenberg Self-Esteem Scale (RSES) [12], and the Basic Self-Esteem Scale (BSES) [13] both assess the degree to which a person’s self-worth remains stable over time [10]. The State Self-Esteem Scale (SSES) was developed by Heatherton and Polivy for use in research on short-term changes in feeling of self-worth [14]. No presently available, valid scale evaluates the feeling of self-worth; however, one item of the 10-item RSES reads: “I feel that I am a person of worth, at least on an equal plane with others” [12]. In addition to RSES, several clinical scales which focus on the assessment of depression have one or two items capturing “worthlessness”, including the Center for Epidemiologic Studies Depression Scale, “I felt I was just as good as other people (reverse scored) [15].

Few publications have investigated menstrual cycles or ovulation and self-worth. Hill and Duranate [16] in two studies measured self-worth (what they called “self-esteem”) across the menstrual cycle by using 10-item RSES [12]. The urine luteinizing hormone (LH) surge was used to detect ovulation in both studies with increased self-worth before ovulation. Given evidence that different research-related experiences could cause variability in self-worth, we hypothesized there could be an interaction between a woman’s feeling of self-worth and her longitudinal patterns of ovulation within the menstrual cycle.

Because self-worth has been an under-examined concept, we performed a literature search including the search terms: “self-worth”, “self-esteem”, “menstrual cycle”, “ovulation,” “luteal phase,” “follicular phase” and any related concepts in healthy premenopausal women. We found no study of feeling of self-worth related to menstrual cycles and valid documentation of ovulation. The following study was designed to describe Feeling of Self-Worth, recorded daily over one-year, and its relationships if any, with menstrual cycle or ovulatory characteristics.

Although progesterone has been thought to only work in the uterus, there is increasing evidence that it acts throughout the body in balance with estradiol [17]. Further, there are now data that progesterone and ovulation increase bone formation [18] and have positive influences on the cardiovascular system [19,20]. In addition, data show that subclinical ovulatory disturbances (SOD, meaning short luteal phases and anovulation within regular cycles, were markedly increased during lockdowns early in the SARS-CoV-2 pandemic in community-dwelling women who were not ill and had not yet been vaccinated [21]. We can also infer that SOD are, like the related and more obvious and influential hypothalamic amenorrhea [22], adaptive, protective and reversible in response to psychosocial, nutritional, physiological or illness-related “threats”. Given this complex evidence that ovulation is associated with an individual’s multi-dimensional well-being, we hypothesized Feeling of Self-Worth would be higher in normally ovulatory cycles and in the luteal compared with the follicular phase.

## Materials and methods

This study is a secondary analysis of menstrual cycle and ovulation-related experiences from the archived, 1- year Prospective Ovulation Cohort (POC) study [1] originally conducted in the mid-1980s at the University of British Columbia (UBC) in Vancouver, British Columbia, Canada [2]. Although the parent study, and the overall evaluation of menstrual cycle experiences both had ethical approval, this sub-analysis, has also been specifically approved by UBC Clinical Research Ethics Board (# H23-02900). On October 10, 2023, from original archived data, the data were accessed for research purposes of this secondary analysis.

### Study population

Participants were comunity dwelling women of any race or ethnicity from Metro Vancouver who were regularly cycling, aged 20–41 years. They provided written informed consent after fully understanding and agreeing to the research. Before enrolment, women were required to have two consecutive, normal-length (21–36 day) and normally ovulatory (luteal phase length ≥ 10 days) cycles by the validated [22,23] Quantitative Basal Temperature© (QBT©) analysis method. Other inclusion criteria were a normal Body Mass Index (BMI) of 18.5–24.9, being in good general physical and emotional health, with a range of physical activity levels from sedentary to training for and running a marathon during the study year. Exclusion criteria were weight loss or gain of 2.5 kg in the last year, cigarette use, clinical androgen excess or Polycystic Ovary Syndrome (PCOS), use of hormonal contraceptives or ovarian hormones within the past six months, working shifts or a student with unpredictable sleeping and awakening hours (which could make the QBT© less reliable), currently pregnant or planning a pregnancy, having an eating disorder, being a compulsive exerciser who would exercise even if injured or unwell, or unwilling/unable to record the Diary©, exercise and first morning temperatures daily, and complete a 7-day diet diary every 3–4 months.

### Study design

This is a prospective 1-year observational study of Menstrual Cycle Diary© (Diary) data (completed in the evening before sleep) by healthy, community dwelling, premenopausal women describing Feelings of Self-Worth. The primary objective was to describe the variability of Feeling of Self-Worth in 53 women and 698 cycles and its association or not with ovulation. In all normally ovulatory cycles with follicular and and normal length luteal phases (≥10 days), we examined Self-Worth in the follicular versus luteal phases. We did the same analysis in all ovulatory cycles (including those with short luteal phases). Other objectives included assessing Feeling of Self-Worth within-woman in the 46 women who experienced both normally ovulatory and ovulatory disturbed cycles between these two cycle types in the whole cycle. We further sought to describe Feeling of Self-Worth and its relationship with other simultaneously recorded Diary experiences and negative moods.

### Study evaluation and measurements

Feeling of Self-Worth was assessed by an ordinal-scale item in the Diary, scored daily as a letter. The letter “U” indicated a participant’s usual feeling with two letters above and two below to allow a graded response. For analysis, these letters were replaced by numbers with the lowest being “1”, the highest “5,” and “usual” Feeling of Self-Worth scored as a “3.”

Enrolled women from the parent study were invited to complete the Menstrual Cycle Diary© (Diary) [3] to record their daily life and menstrual cycle-related experiences as an optional part of the original protocol. She would start the Diary© with the first day of flow. The Diary was used to track cycle length and record first morning temperatures. Diary data may also help women to perceive connections between their feelings and experiences [24] and have been reported for negative moods [25], fluid retension [26], interest in sex [27], and menstrual cramps [28].

To assess Feeling of Self-Worth related to ovulation and its characteristics, we used the reliable, twice-validated Quantitative Basal Temperature© (QBT©) method. This was accomplished by the first morning oral, waking temperature, collected via a provided single-batch mercury low-reading thermometer, recorded on a line at the bottom of the Menstrual Cycle Diary©. We asked women to record their running exercise as minutes/day (on a separate form), as well as their minutes of other moderate exercise such as aerobics, swimming, skiing, fast walking, playing tennis, and dancing.

### Statistical analysis

We initially excluded cycles missing >33% of days of scoring Feeling of Self-Worth data. Then, we assessed all data for its distribution and used appropriate parametric statistical methods for normally distributed data, and non-parametric for non-normally distributed datasets. Baseline demographic, anthropometric, reproductive, and exercise variables were also described using the mean or median and SD or range, depending on the distribution. Feeling of Self-Worth for the whole cohort was divided into two categories as equal to, above or below to the median (≥ 3). Given the ordinal nature of the Diary notations, within-woman analyses were conducted using Wilcoxon Paired Signed Rank Test. We used the same test to analyze Feeling of Self-Worth related to within-woman ovulatory characteristics in the follicular and luteal phases of all cycles that were normally ovulatory (luteal length [LL] ≥10 days) and also in *all* ovulatory cycles (including those with short luteal phases, LL < 10 days). To determine the relationships of Feeling of Self-Worth with all other variables in the Dairy, we used Principal Component Analysis with varimax rotation and accepted variable loading of ≥ 0.3 as important. We considered a *P* value of <.01 as likely meaningful given multiple comparisons. We performed all analysis with SPSS version 29 (IBM Corp. 2022. IBM SPSS Statistics for Windows, New York).

## Results

**Fig 1:**
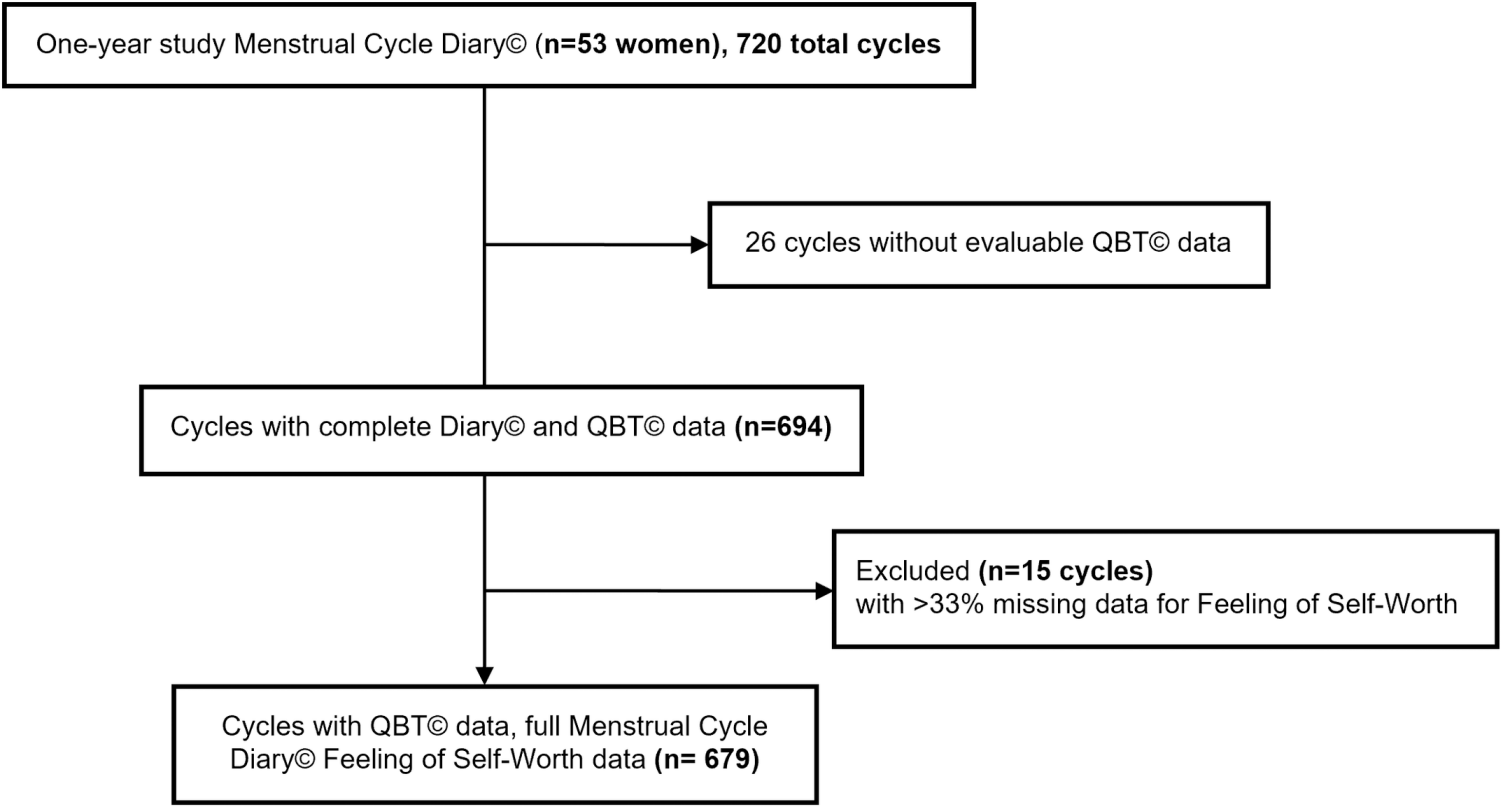
This Consort-like diagram shows the menstrual cycles of 53 participants from the original one-year Prospective Ovulation Cohort study to describe all sufficient Feeling of Self-Worth recordings, and assessment of ovulation by Quantitative Basal Temperature©.

Fifty-three of the completing 66 women in the original 1-year Prospective Ovulation Cohort study had kept ≥ eight cycles of the optional Diary© data (mean of 13). These 53 women’s data included a total of 720 menstrual cycles with 694 cycles having valid QBT© ovulation data and 15 cycles being excluded for having more than 33% of days missing data for Feeling of Self-Worth. Fig 1 shows the flow of participants through this study.

**Fig 2:**
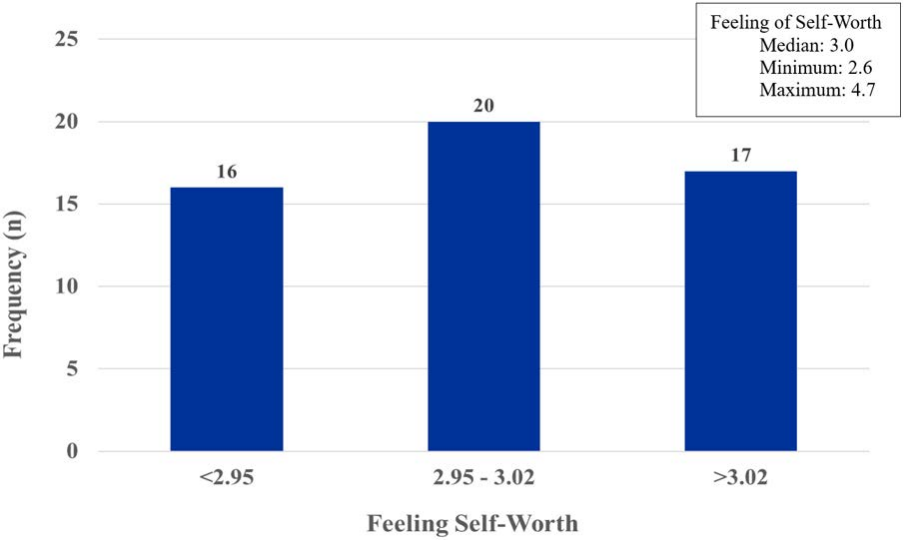
Frequency distribution of one-year feeling of self-worth in 53 women* (698 cycles) from the Prospective Ovulation Cohort (POC) in those with or without Ovulation Data. ^a^ This dataset includes 698 cycles. From a total of 720 cycles in the POC, we have excluded 22 menstrual cycles with >33% missing data for the Feeling of Self-Worth variable.

To provide information about the whole cohort’s Feeling of Self-Worth evaluation, all cycles with sufficient data, whether or not they had information on ovulation status (QBT© data) were included as shown in Fig 2.

In Table 1 the baseline characteristics of the 53 women in this investigation were documented by median split of Feeling of Self-Worth. None of the variables differed between the two groups. Participants averaged 34 years of age and had a BMI of 22. Some of the participants had used combined hormonal contraceptives (CHC) in the past or been pregnant for varying numbers of months. This was a rather active cohort with a mean of 82 minutes of mild-moderate exercise per cycle plus 311 minutes of running each cycle.

**Table 1:** Baseline demographic, anthropometric and reproductive variables in investigation of feeling of self-worth (by the sample median) from the menstrual cycle diary© daily records over about a year in the prospective ovulation cohort.

		Feeling of Self-Worth		
		<3	≥ 3	
Characteristic	Entire Cohort, n = 53 (679 cycles)	n = 26	n = 27	*P-*value
Mean (SD)				
Age (years)	33.9 (5.5)	33.5 (6.2)	34.4 (4.9)	.56^ᵃ^
Height (cm)	162.5 (6.4)	162.2 (6.7)	162.9 (6.2)	.69^ᵃ^
Weight (kg)	58.1 (6.7)	58.4 (7.5)	57.8 (6.1)	.76^ᵃ^
Body Mass Index (kg/m²)	22.0 (2.1)	22.2 (2.1)	21.8 (2.1)	.53^ᵃ^
Age at menarche (years)	11.5 (1.2)	11.4 (1.3)	11.5 (1.1)	.68^ᵃ^
Cycle Length (days)	28.1(2.3)	28.0 (2.2)	28.2 (2.3)	.76^ᵃ^
Median (Range)				
Moderate exercise (minutes/cycle) ^c^	82 (0.0-854)	73 (0.0-854)	82 (0.0-477)	.74^b^
Running exercise (minutes/cycle)	311 (0.0-1573)	390 (0.0-1573)	290 (0.0-1065)	.30^b^
Lifetime CHC use^d^ (months)	33.0 (0.0-156.0)	28.0 (0.0-156.0)	39.0 (0.0-143.0)	.60^b^
Lifetime months of pregnancy	3.0 (0.0- 40.0)	2.5 (0.0-40.0)	3.0 (0.0-30.0)	.40^b^

ᵃ One way ANOVA;

^b^ Independent-Samples Mann-Whitney U Test

^c^ Any mild-moderate exercise other than running such as aerobics, swimming, skiing, walking, playing tennis, dancing;

^d^ CHC = Combined Hormonal Contraceptives

Table 2 illustrates the within-women analysis of Feeling of Self-Worth in the follicular and luteal phases of ovulatory cycles. The top section shows Feeling of Self-Worth across follicular and luteal phases in all *normally* ovulatory cycles in which there was no difference between the follicular versus the luteal phase values (*P* = .18). However, when *all *ovulatory** cycles (that included those with short luteal phases) were examined by phases, the follicular phase showed slightly higher Feeling of Self-Worth (*P* = .01) than did the luteal phase.

**Table 2:** Feeling of self-worth by cycle phases within women in all normally ovulatory versus all ovulatory cycles, plus, within-woman comparing whole cycle data in all normally ovulatory versus subclinical ovulatory disturbed cycles within the 46 women with normally ovulatory, short luteal and anovulatory cycles in the 1-year prospective ovulation cohort.

Whitin Women Feeling of Self-Worth by Ovulatory Cycle Phases in All Ovulatory versus only Normally Ovulatory Cycles								
	**Normally Ovulatory Cycles** (^*^LL ≥ 10 days)				**All Ovulatory Cycles** Normally Ovulatory (*LL ≥ 10 days) & Short Luteal Phase Cycles (*LL < 10 days)			
	**53 women, 483 cycles**				**53 women, 661 cycles**			
	**Follicular Phase**	**Luteal Phase**	Mean Positive Rank	*P value*	**Follicular Phase**	**Luteal Phase**	Mean Positive Rank	*P* value
	Median (Min – Max)							
**Feeling of** **Self-Worth**	3.00 (2.59-4.45)	3.00 (2.25-4.56)	23.85	.18ᵃ	3.00 (2.61-4.63)	2.99 (2.54 −4.71)	23.73	.01ᵃ
**Whole Cycle Feeling of Self-Worth Scores Within-Woman in Normally Ovulatory vs Ovulatory Disturbed Cycles**								
	**Normally Ovulatory Cycles**				**Ovulatory Disturbed Cycles**			
	46 women, 401 cycles				46 women, 196 cycles			
	**Follicular and Luteal/Premenstrual Phases Combined**						Mean Positive Rank	*P* value
	Median (Min – Max)							
**Feeling of** **Self-Worth**	2.99 (2.59-4.51)				3.00 (2.64-5.00)		25.12	.03^ᵃ^

* LL: Luteal length; Normally ovulatory LL by Quantitative Basal Temperature© = ≥10 days; Short Luteal Phase Cycles have LL < 10 days.

ᵃ Wilcoxon Paired Signed Rank Test

Forty-six of the 53 women in this cohort, despite consistently normal cycle lengths, experienced both normally ovulatory and ovulatory disturbed cycles (meaning with short luteal phases or anovulation). Thus, at the bottom of Table 2, a within-woman analysis in 46 women in all follicular and luteal/premenstrual phases of the cycle by normally ovulatory versus ovulatory disturbed cycles, showed that there was a slightly higher Feeling of Self-Worth in ovulatory disturbed vs normally ovulatory cycles (*P* = .03).

### Feeling of self-worth relationship with other menstrual cycle diary© experiences

To understand the relationship of the 18 Diary variables with Feeling of Self-Worth as well as to reduce to fewer “factors,” we performed Principal Components Analysis (PCA). **Table 3** summarizes the results of our PCA analysis that included five factors. The construct of Feeling of Self-Worth was positively loaded on the Interest in Sex Factor along with the Feeling of Energy variable. The Interest in Sex Factor accounted for 9.0% of the total variance. On the other hand, the Feeling of Self-Worth variable also significantly but negatively loaded on the Negative Mood Factor.

**Table 3:** Principal component analysis of all menstrual cycle diary© items to assess factor loading of “feeling of self-worth” All loadings ≥ 0.3 were listed.

Factors (56.8% of Variance)					
	Negative Moods (14.2%)	Menstruation- Related (11.9%)	Breast & Fluid (11.5%)	Physical Discomforts (10.2%)	Interest in Sex (9.0%)
Feeling of Anxiety	0.778				
Feeling of Frustration	0.730				
Feeling of Depression	0.705				
Outside Stresses	0.688				
#Pads & Tampons		0.906			
Qualitative Flow (0–4)		0.900			
Cramps		0.580		0.325	
Breast Sizer			0.859		
Breast Tenderness			0.796		
Fluid Retention			0.582	0.392	
Appetite			0.397		
Headache				0.642	
Constipation				0.593	
Sleep-Disturbance				0.580	
Interest in Sex					0.690
Feeling of Energy					0.675
Feeling of Self-Worth	−0.400				0.651

n = 53 women with 698 Cycles over ~12 months.

Extraction Method: Principal Component Analysis; Rotation Method: Varimax with Kaiser Normalization. Rotation converged in 6 interactions.

To further investigate and illustrate the relationship of Feeling of Self-Worth with menstrual cycle phases, mean data from all ovulatory cycles (n = 661) were aligned to the day of ovulation (QBT© temperature shift day) and shown with their 95% confidence intervals.

**Fig 3:**
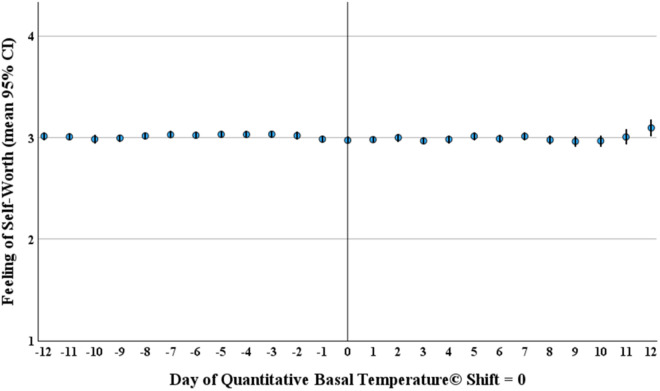
Mean feeling of self-worth per ovulatory menstrual cycle day (95% ci) in 53 women (*661 normally ovulatory and short luteal phase cycles*) from the prospective ovulation cohort, all oriented to ovulation by quantitative basal temperature©.

When we plotted Feeling of Self-Worth across all ovulatory cycles oriented to the day of ovulation by QBT© a very tight distribution was illustrated as shown in Fig 3.

## Discussion

In this healthy cohort of initially normally menstruating and ovulating premenopausal women, we showed that Feeling of Self-Worth did not relate to baseline anthropomorphic, reproductive or exercise variables and that results showed it to be a stable trait with results tightly clustered around a woman’s usual feeling. To our knowledge this is the first study to investigate women’s daily Feeling of Self-Worth related to menstrual cycles and ovulation. Our study showed there was no consistent difference between the follicular and luteal phase records of Feeling of Self-Worth. However, Feeling of Self-Worth was slightly higher, in within-woman analysis, in ovulatory disturbed than in normally ovulatory cycles, which was opposite to our hypothesis. Also, Feeling of Self-Worth was slightly higher in the follicular than the luteal phase when including both normally ovulatory and short luteal phase cycles. Again, this was not what we predicted. In analysis of only *normally* ovulatory cycles, however, there was no significant difference between the follicular and luteal phases. In addition, these data show that Feeling of Self-Worth was closely related to the positive feelings of interest in sex, and energy as we previously showed [27]. Feeling of Self-Worth was also negatively related to depression, anxiety and frustration.

Research has previously shown that women’s menstrual cycle may have important influences on how a woman thinks, feels, and behaves [29]. Reviewing the literature, we found few publications had investigated menstrual cycles/ovulation and self-worth. One that did study menstrual cycles, documented “ovulation” erroneously solely by cycle day [30]. There was also a paper showing increased self-worth during the days before the midcycle LH surge in an ovulatory cycle [16]. It is of note that an LH surge may occur and not be followed by ovulation and luteal phase progesterone levels, especially in perimenopausal women [31]. Overall, we found no evidence of ovulation-related variation self-worth.

Other research supports our negative association of Feeling of Self-Worth with negative moods including depression. To diagnose major depressive episode presence of five out of nine criteria, including worthlessness is required [9]. A study in women in their late teens showed that low self-esteem made women vulnerable to depression in face of problematic life events and stresses [32]. Rosenberg et al. [33] also showed that the relationship between self-esteem and depression was bidirectional. Authors, by using six items of the 10- item Rosenberg Self-esteem scale (RSES) in a secondary analysis of a data collected at two points in time, showed that there was a bidirectional relationship between self-esteem and depression. At both times, self-esteem and depression were correlated with r values of −.45 and −.58 [33].

Our study has several limitations. First of all, they are secondary analysis of data collected for another purpose. In addition, they rely solely on self-report, although their reliability is strengthened by the long duration of daily records. Only fifty-three individual women contributed data to this database. Finally, we did not use a validated self-worth tool. Despite these limitations, however, our study also has several strengths. These include: we used a twice-validated Quantitative Basal Temperature© (QBT©) analysis method to determine both ovulation day and the luteal phase length; participants were from the community, which makes more valid generalization of the results. Most important is that we have collected and analyzed an average of a year’s worth of daily data per person.

## Conclusion

Our comprehensive, prospective study of Feeling Self-Worth recorded daily across the menstrual cycle showed no consistent relationship with any baseline variable nor with ovulation, cycle phases or changes across the menstrual cycle. Therefore, small changes around a woman’s “usual” Feeling of Self-Worth are likely related to other daily, non-recorded experiences which are unique to each person. We found small associations of Feeling of Self-Worth and other positive experiences such as interest in sex and feeling of energy. Self-Worth also was negatively correlated with mood variables such as depression. This comprehensive evidence suggests that Feeling of Self-Worth is a trait that is rather stable within an individual and is minimally if at all related to women’s menstrual cycles and ovulation. More research continues to be needed.
